# Supplementary figures and images for: ER export via SURF4 uses diverse mechanisms of both client and coat engagement
Source: J Cell Biol. 2024 Nov 12;224(1):e202406103. doi: 10.1083/jcb.202406103 (PMC11557686; doi:10.1083/jcb.202406103)

**A**

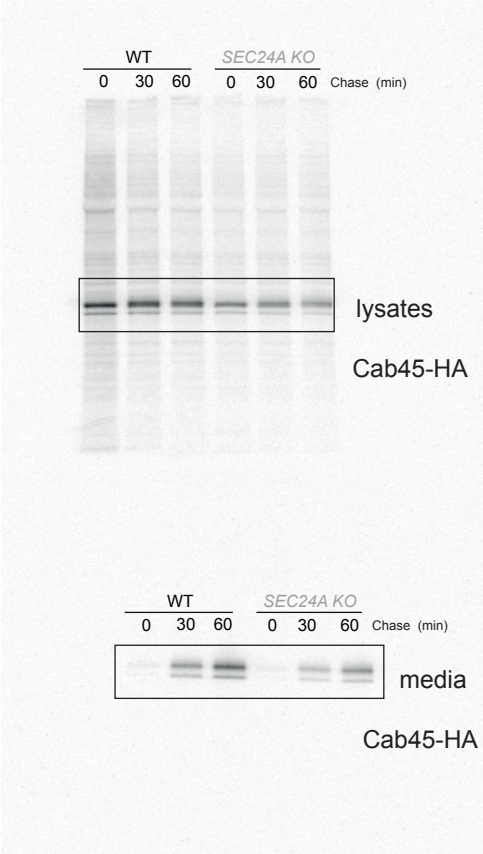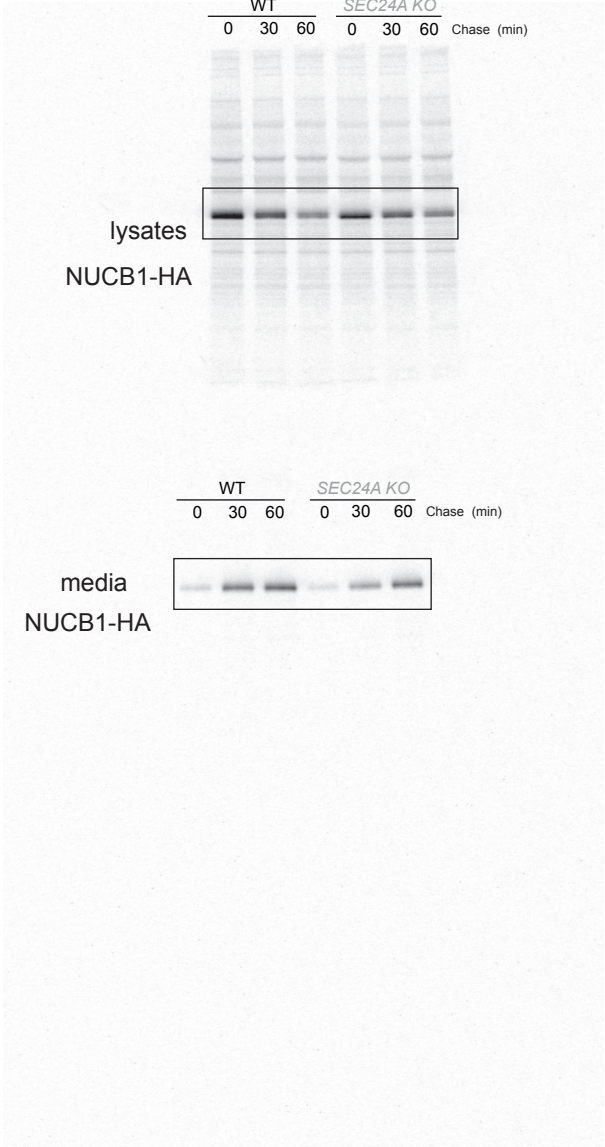

**B**

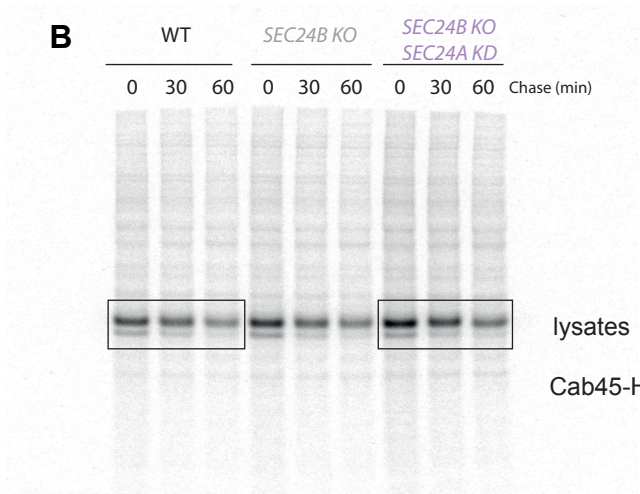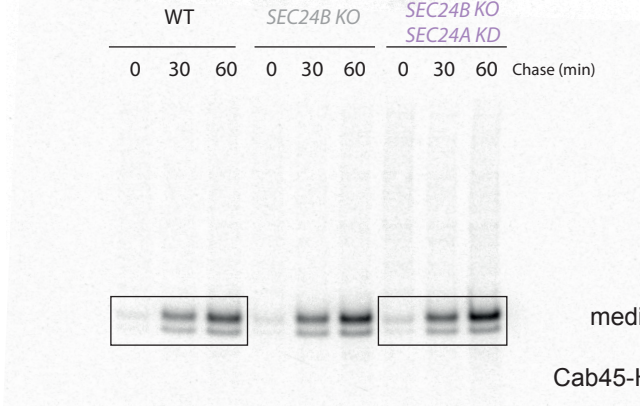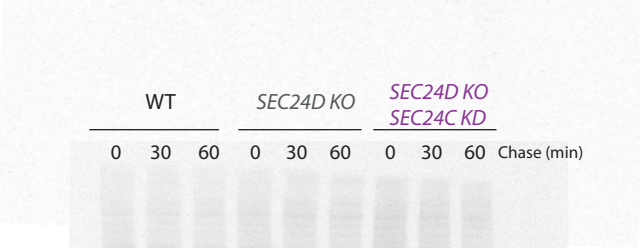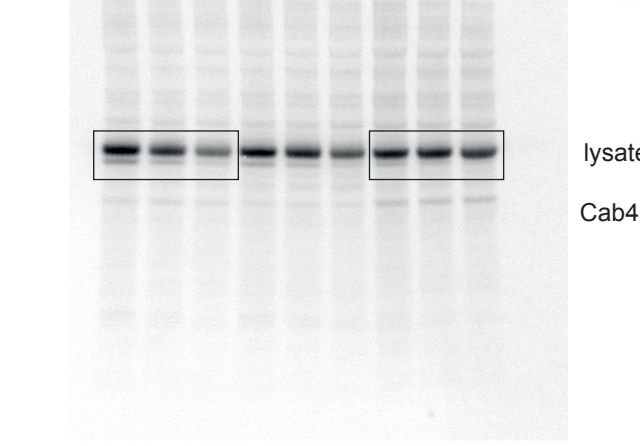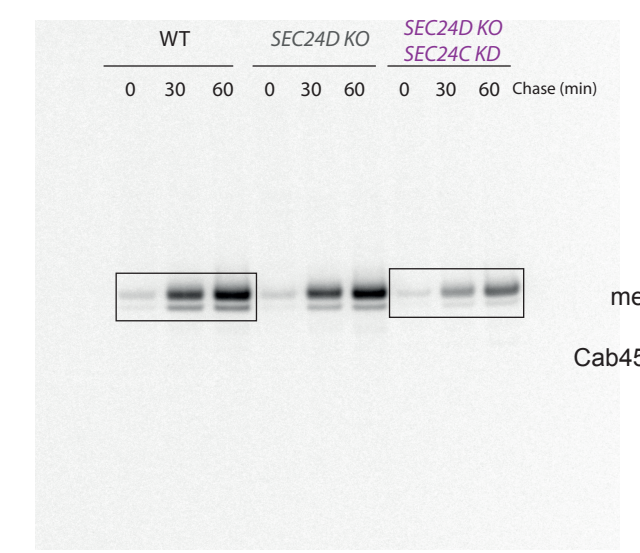

Supplement: SourceData F1 — is the source file for Fig. 1. [file JCB_202406103_SourceDataF1.pdf]

A

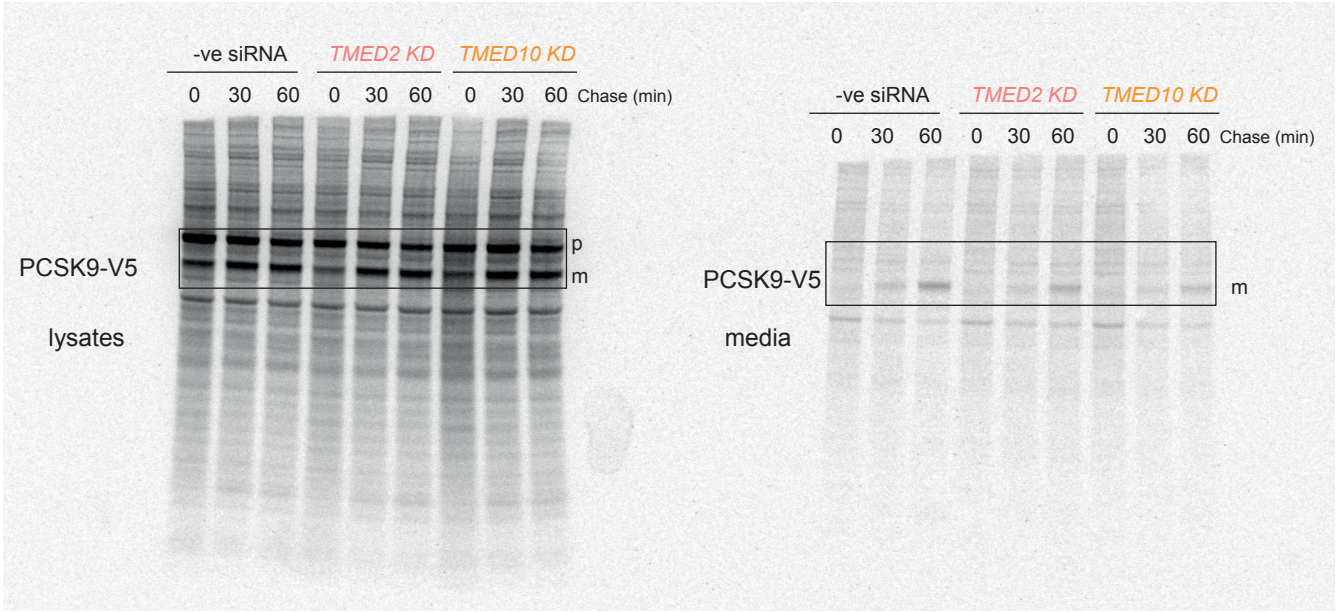

D

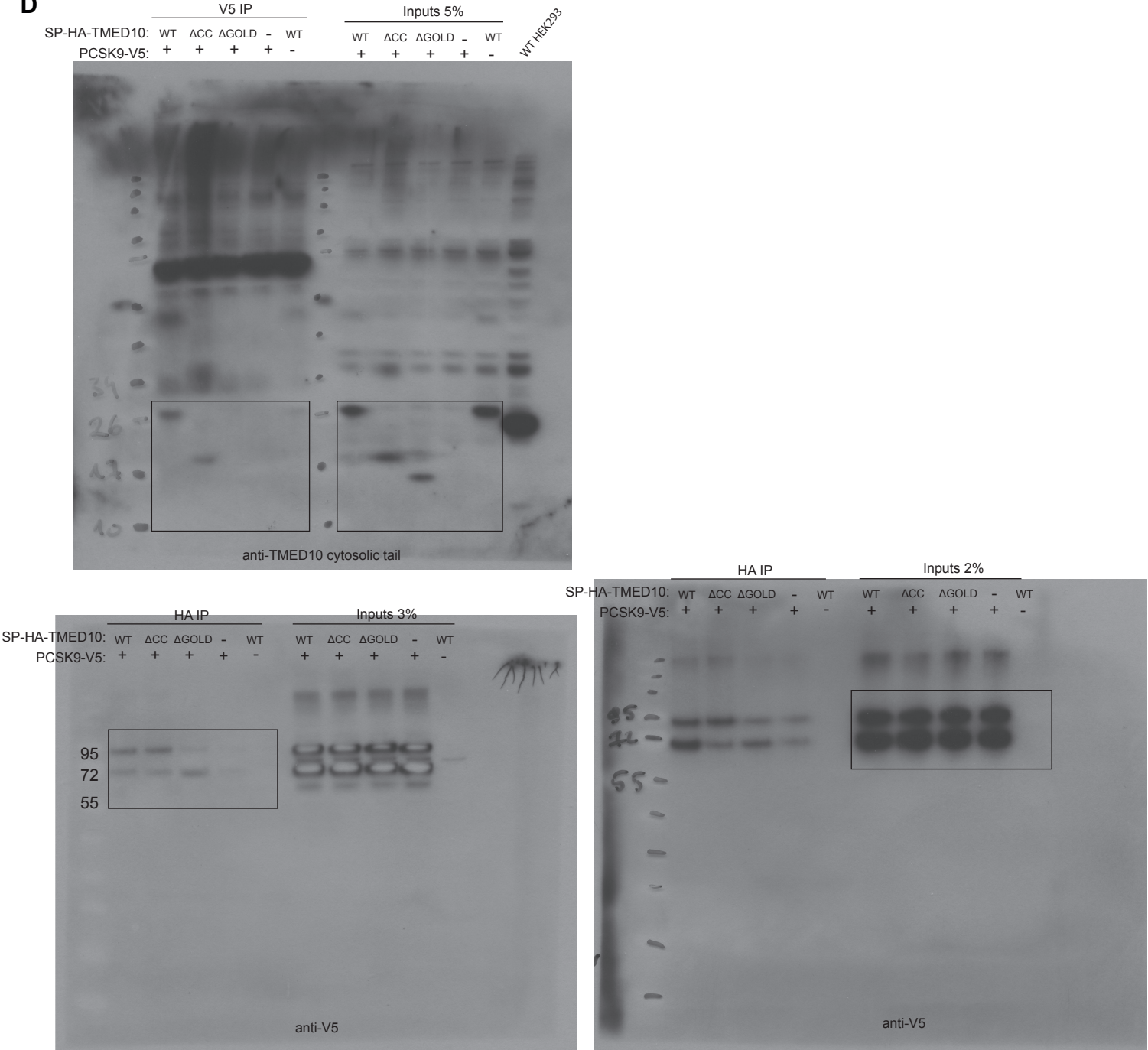

Supplement: SourceData F3 — is the source file for Fig. 3. [file JCB_202406103_SourceDataF3.pdf]

TMED2 KD

TMED10 KD

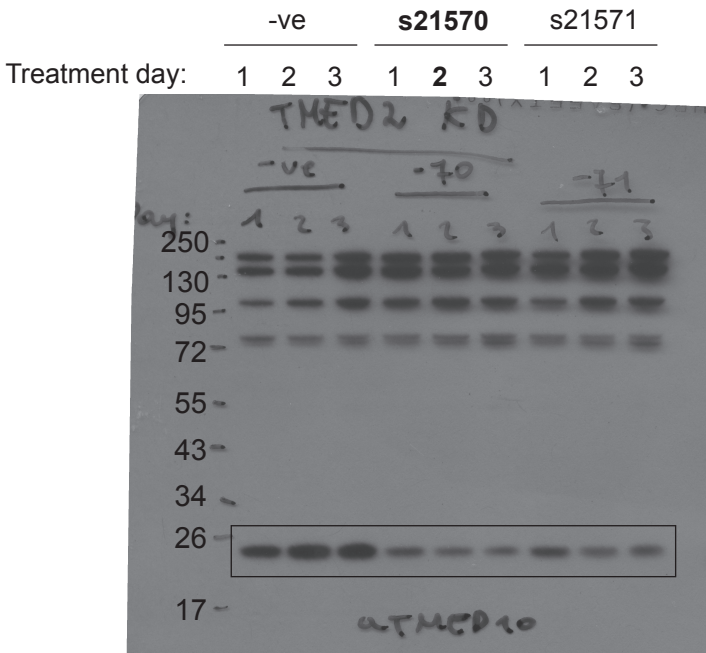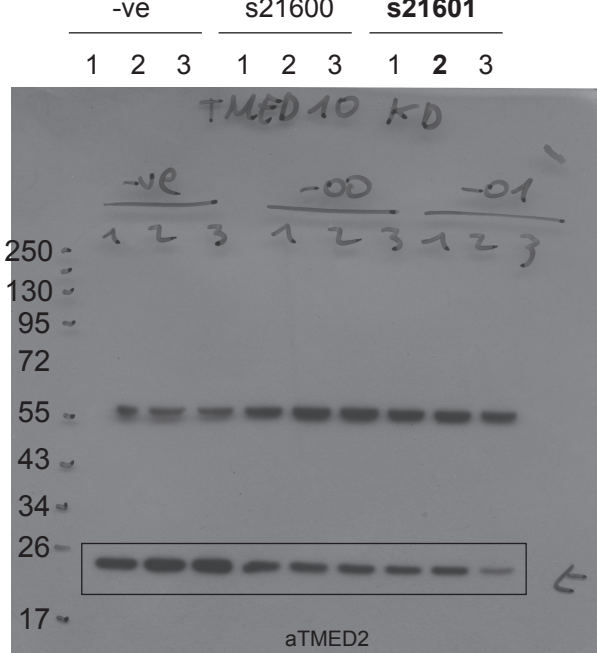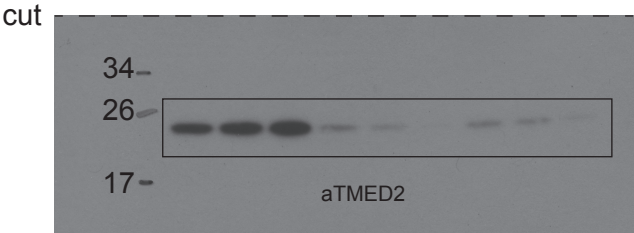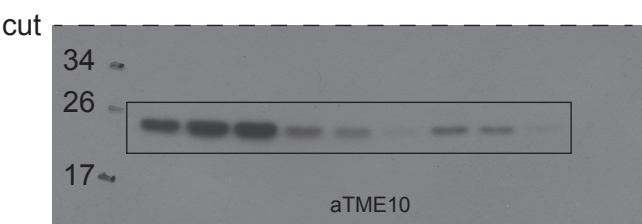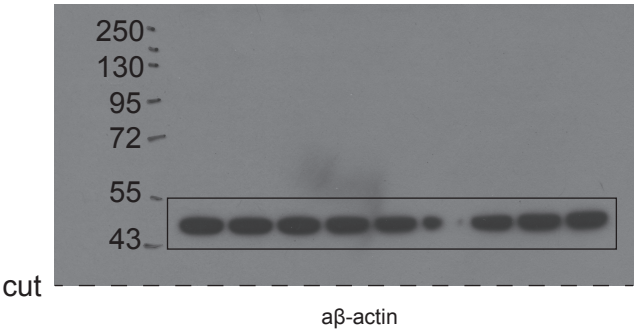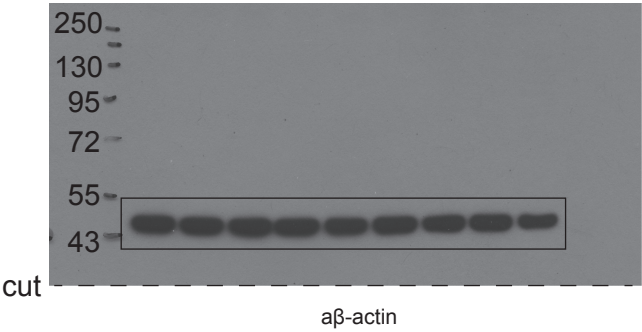

C

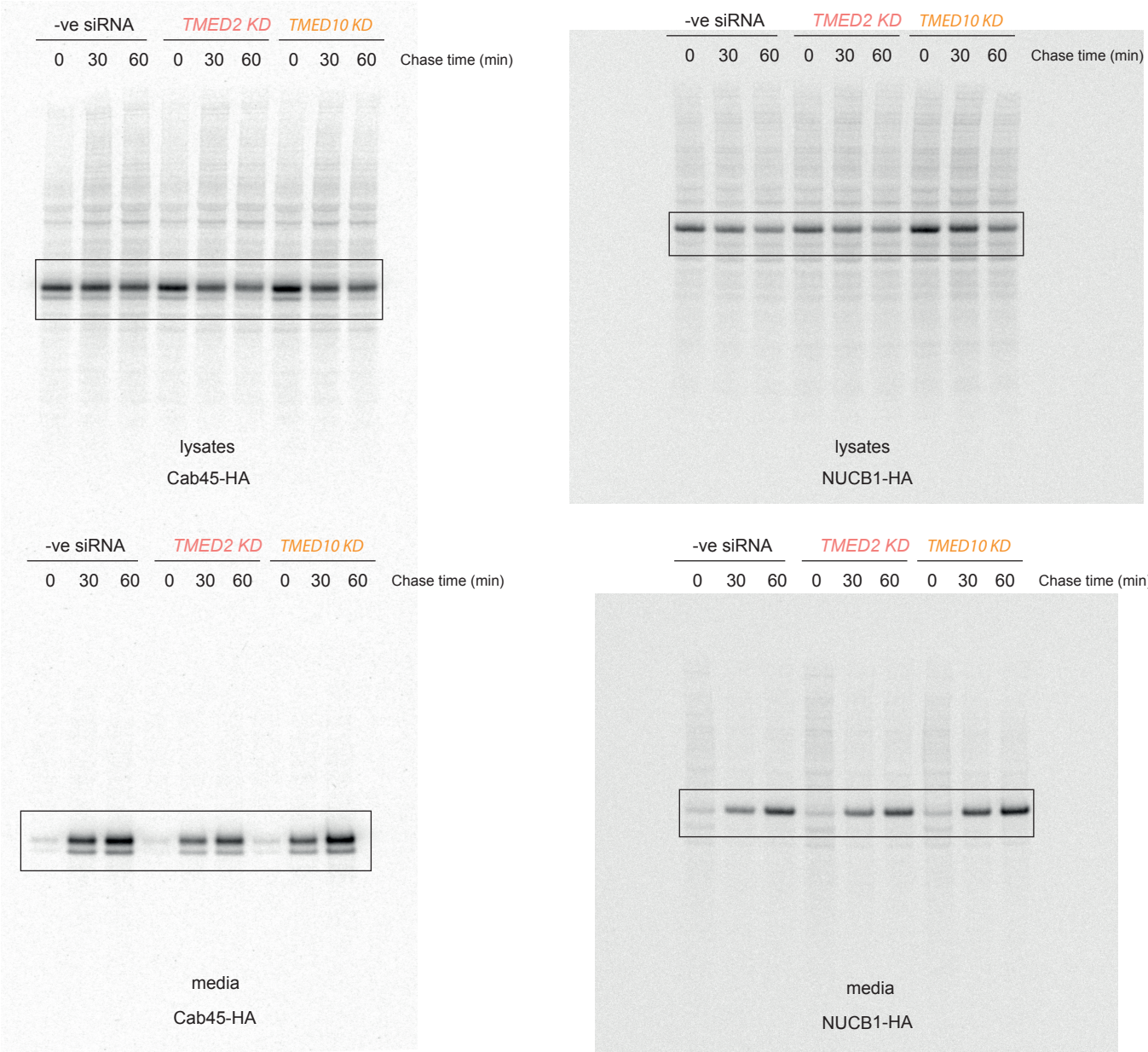

F

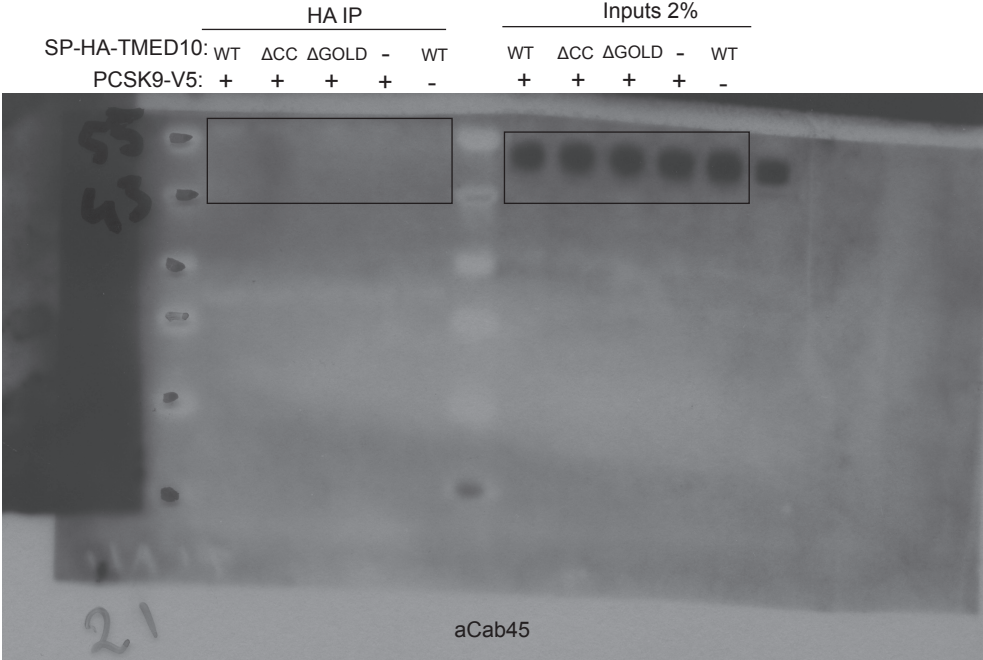

Supplement: SourceData FS3 — is the source file for Fig. S3. [file JCB_202406103_SourceDataFS3.pdf]
