# Supplementary material for: ER export via SURF4 uses diverse mechanisms of both client and coat engagement
Source: J Cell Biol. 2024 Nov 12;224(1):e202406103. doi: 10.1083/jcb.202406103 (PMC11557686; doi:10.1083/jcb.202406103)

SourceDataF4

B

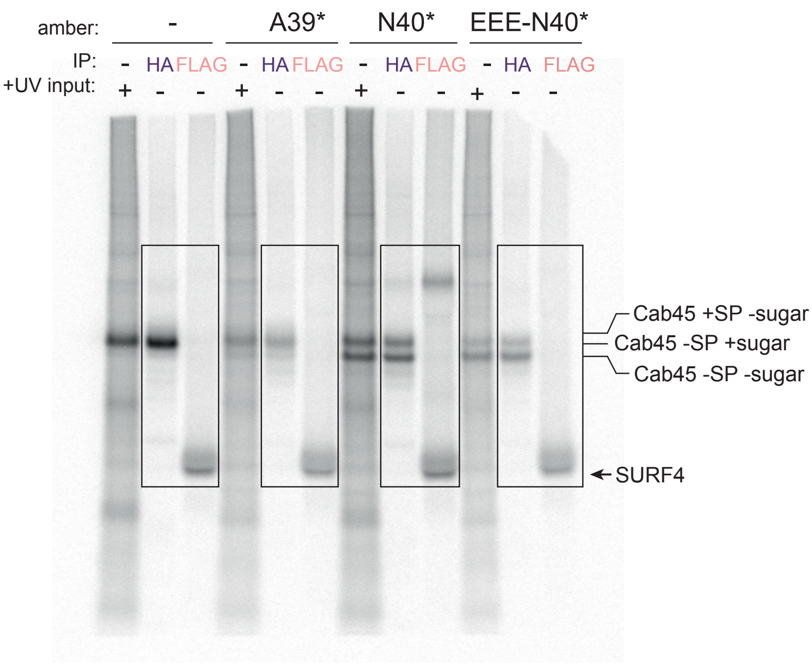

molecular weights in the main figures were obtained by aligning with the stained coomassie gel

D

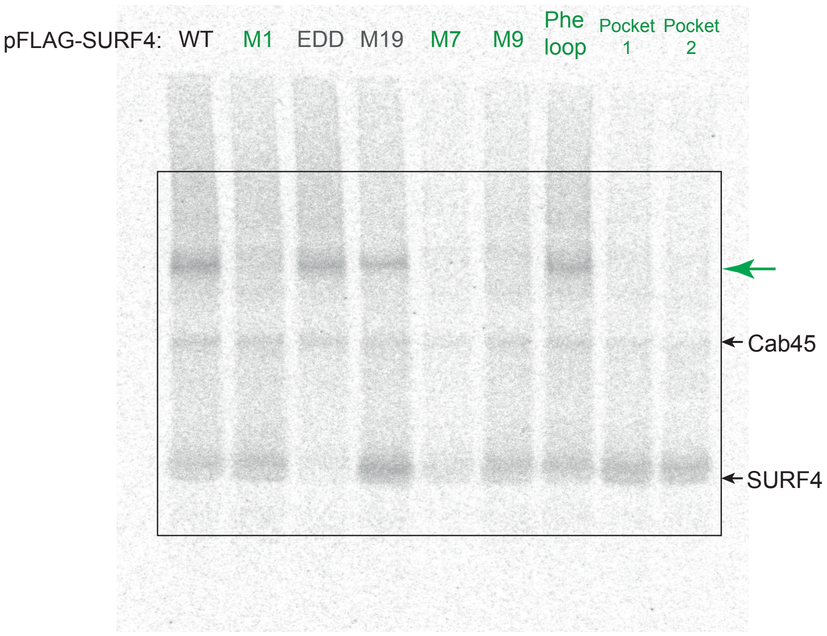

Supplement: SourceData F4 — is the source file for Fig. 4. [file JCB_202406103_SourceDataF4.pdf]
