# Supplementary material for: ER export via SURF4 uses diverse mechanisms of both client and coat engagement
Source: J Cell Biol. 2024 Nov 12;224(1):e202406103. doi: 10.1083/jcb.202406103 (PMC11557686; doi:10.1083/jcb.202406103)
Supplement: SourceData F5 — is the source file for Fig. 5. [file JCB_202406103_SourceDataF5.pdf]

SourceDataF5

A

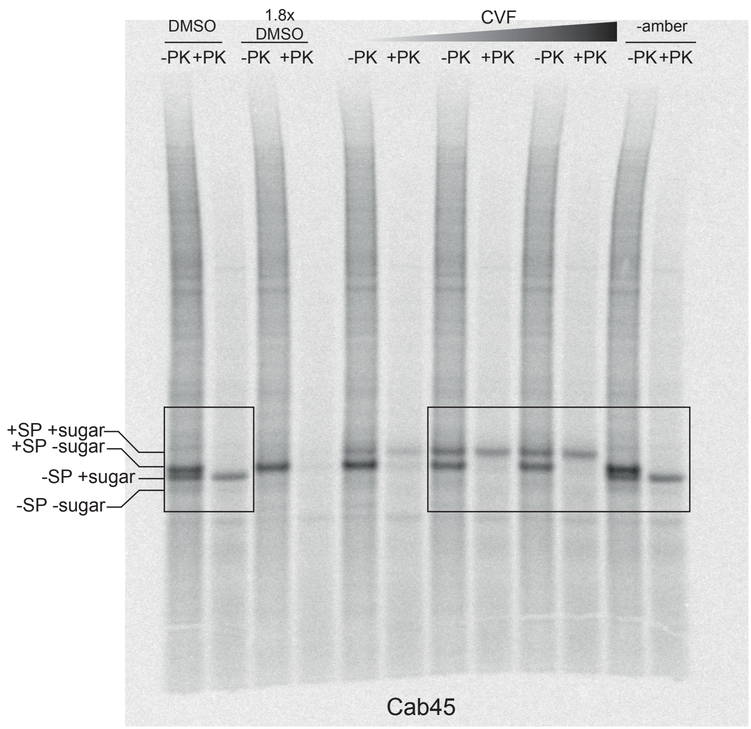

B

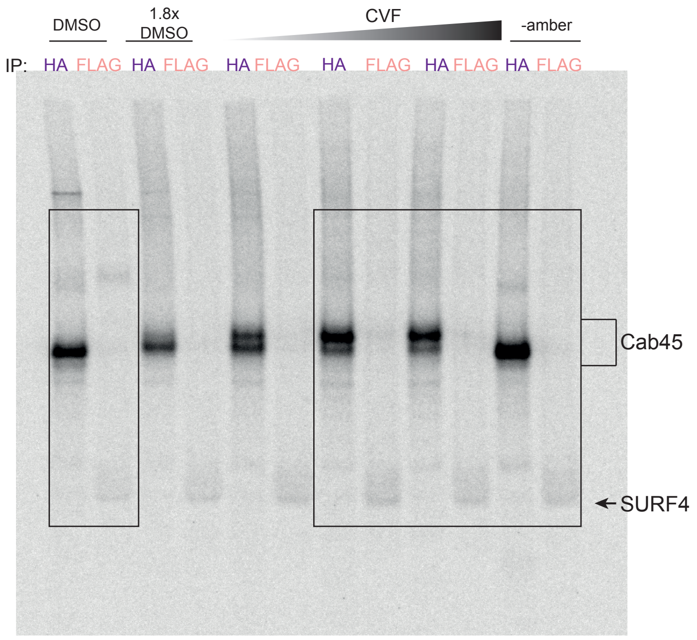

C

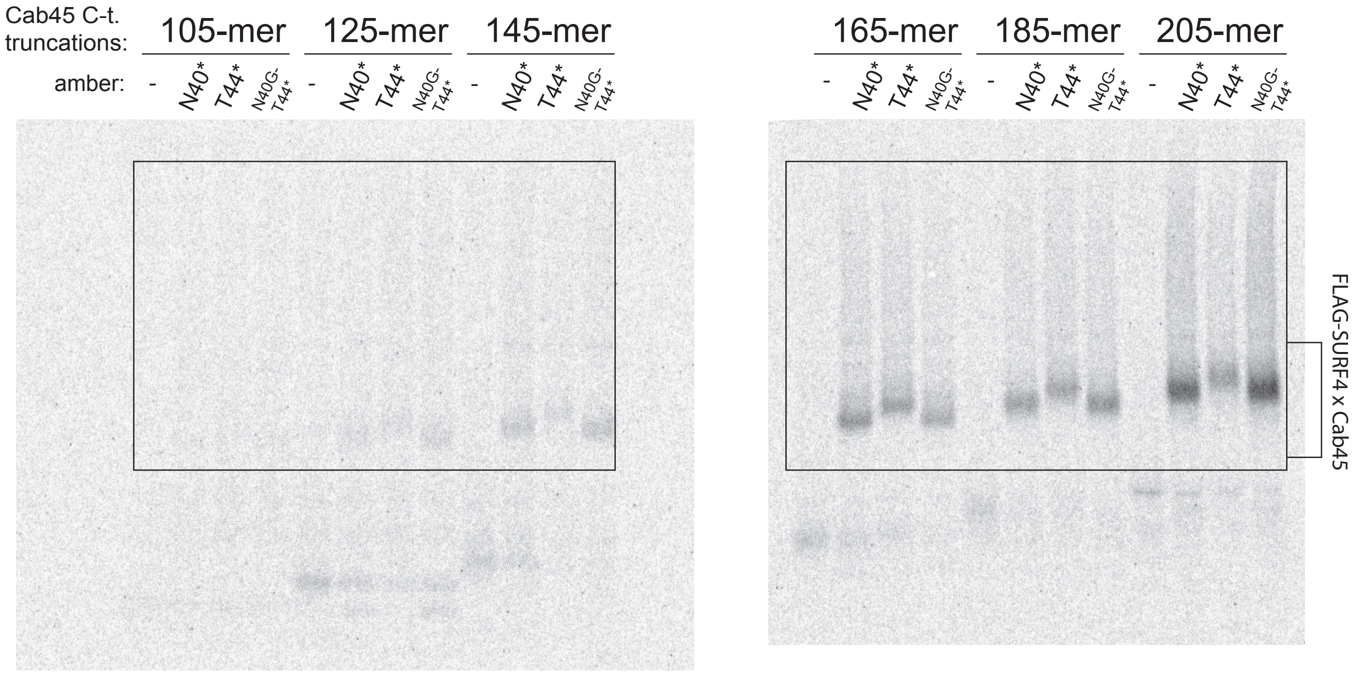

molecular weights in the main figure were obtained by aligning with the stained coomassie gel
