# Supplementary material for: ER export via SURF4 uses diverse mechanisms of both client and coat engagement
Source: J Cell Biol. 2024 Nov 12;224(1):e202406103. doi: 10.1083/jcb.202406103 (PMC11557686; doi:10.1083/jcb.202406103)

SourceDataFS1A

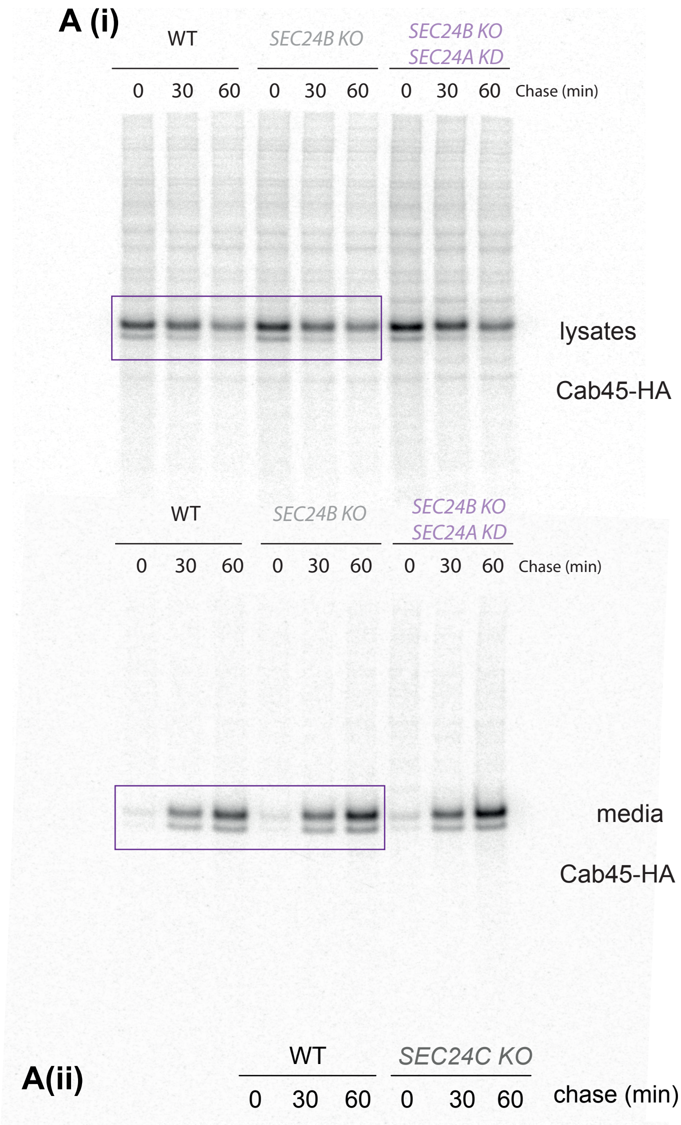

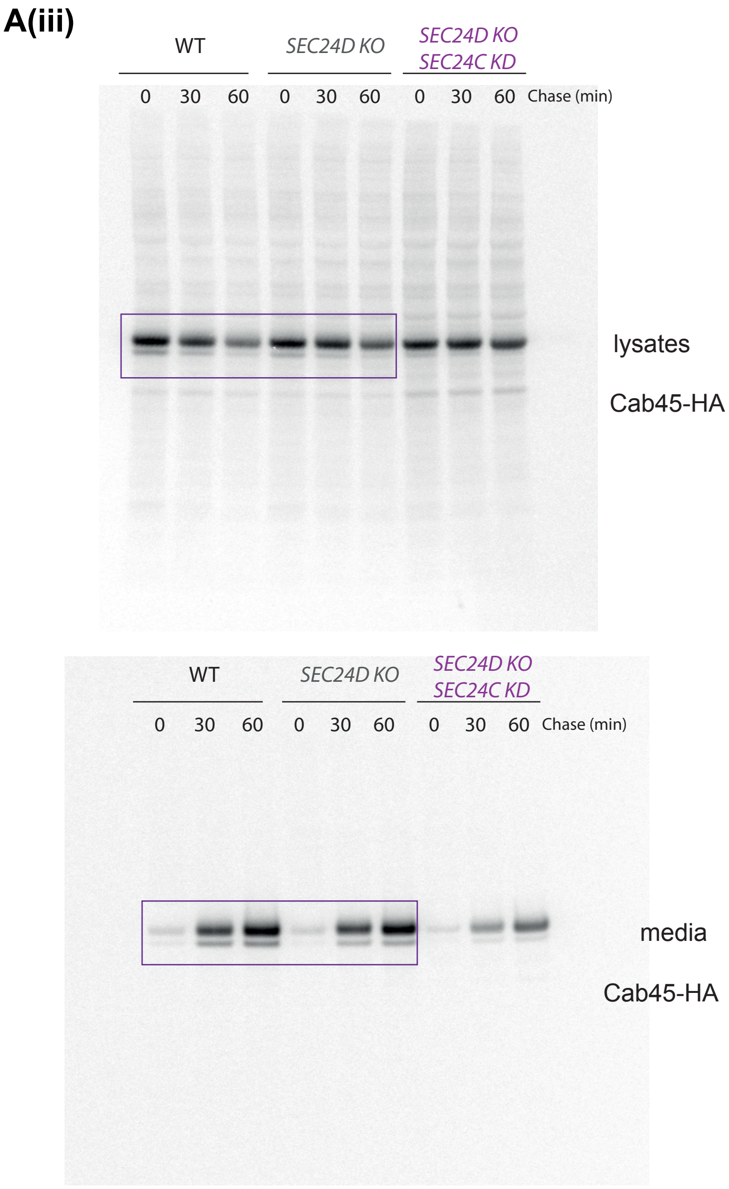

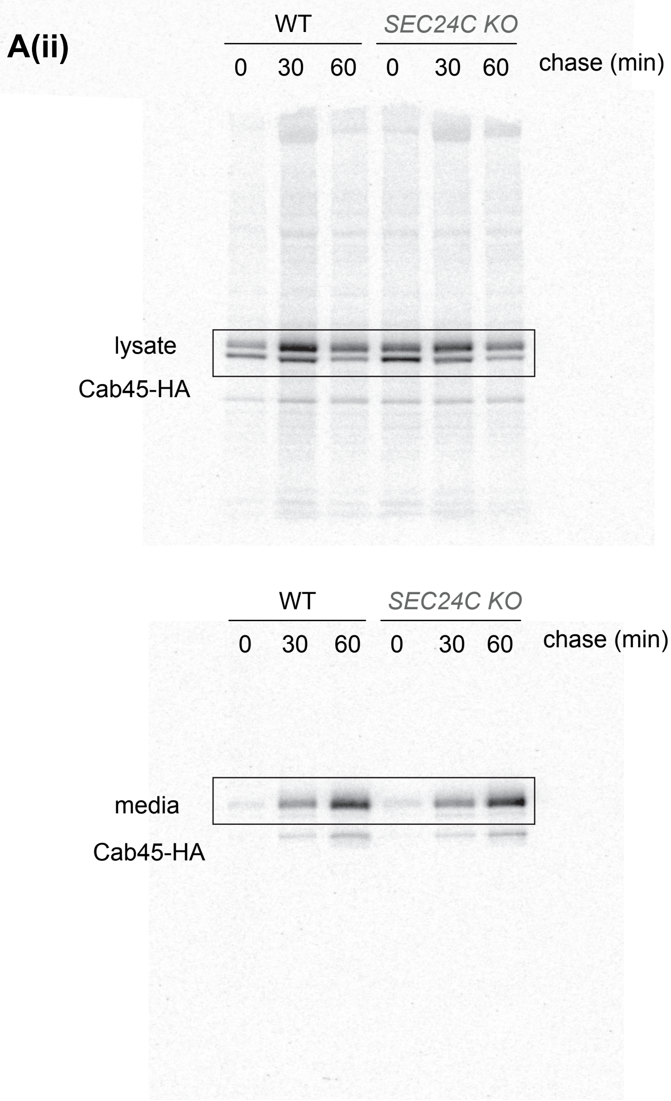

SourceDataFS1B

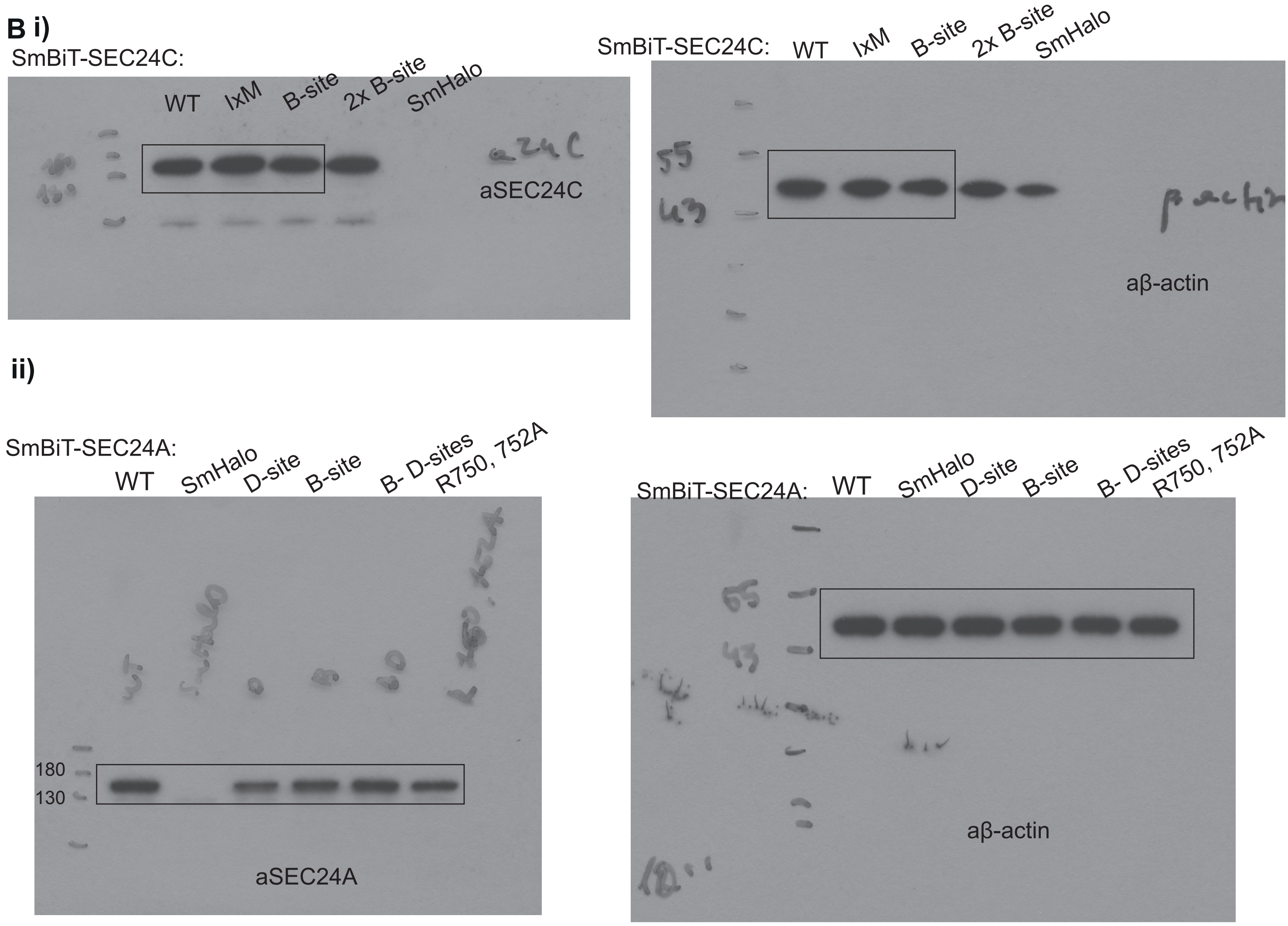

SourceDataFS1D

D

SURF4 KO + pFLAG-SURF4: - FLAG-SURF4 empty M1 M2 M3 M5 M7 M8 M9 Phe loop (M10) M11 DE (M12) EW (M13) M15 M16 M17 M18

long exposure

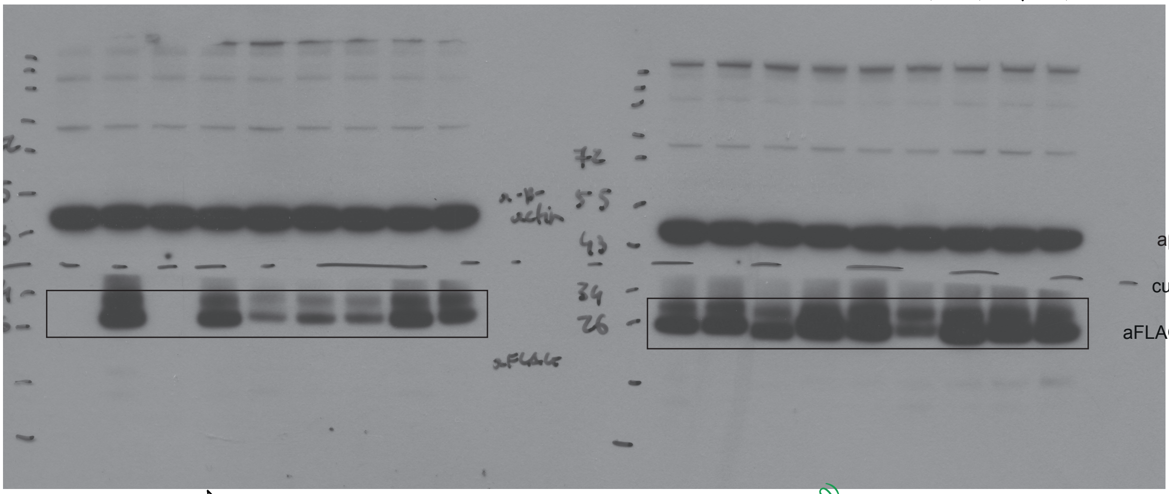

aβ-actin, lysates
cut membrane
aFLAG(SURF4), lysates

SURF4 KO + pFLAG-SURF4: - FLAG-SURF4 empty M1 M2 M3 M5 M7 M8 M9 Phe loop (M10) M11 DE (M12) EW (M13) M15 M16 M17 M18

short exposure

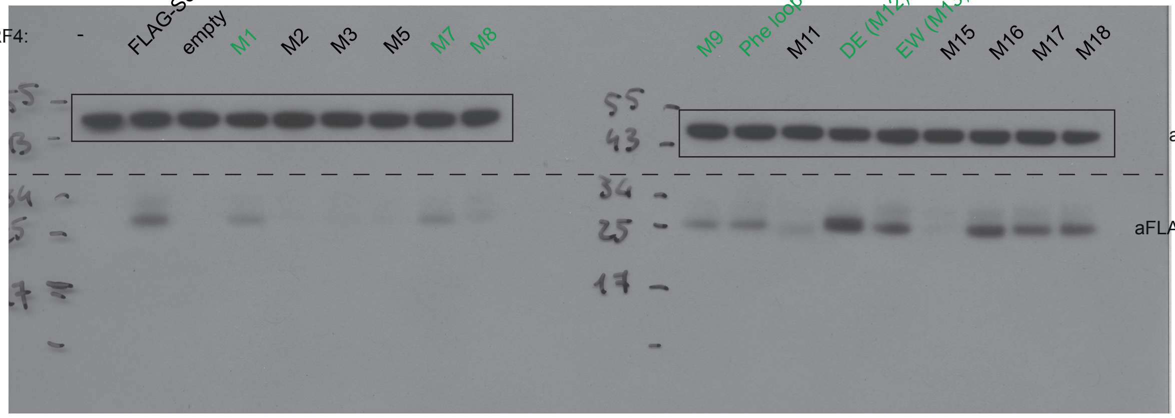

aβ-actin, lysates
cut membrane
aFLAG(SURF4), lysates

SURF4 KO + pFLAG-SURF4: - FLAG-SURF4 empty M1 M2 M3 M5 M7 M8 M9 Phe loop (M10) M11 DE (M12) EW (M13) M15 M16 M17 M18

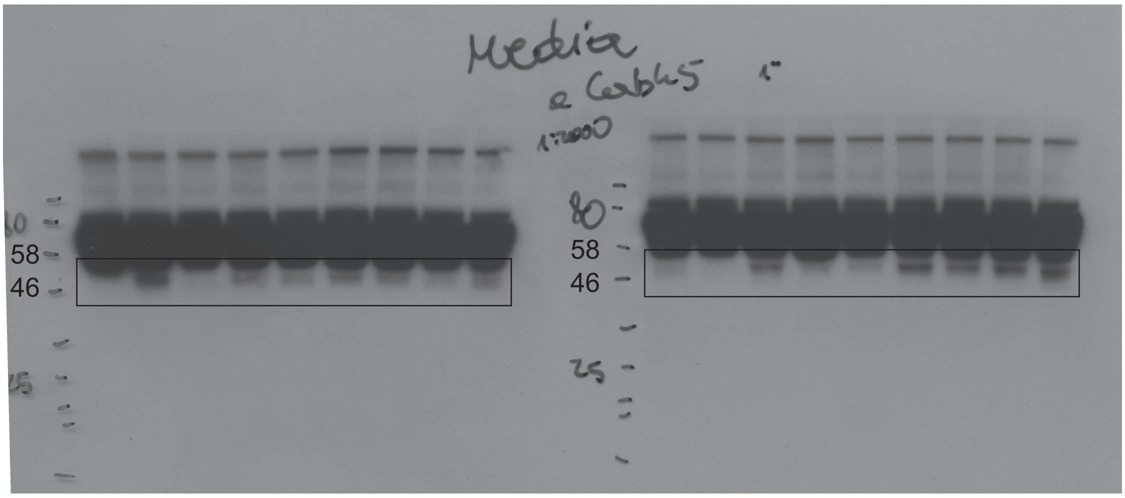

aCab45, media

SourceDataFS1E

E

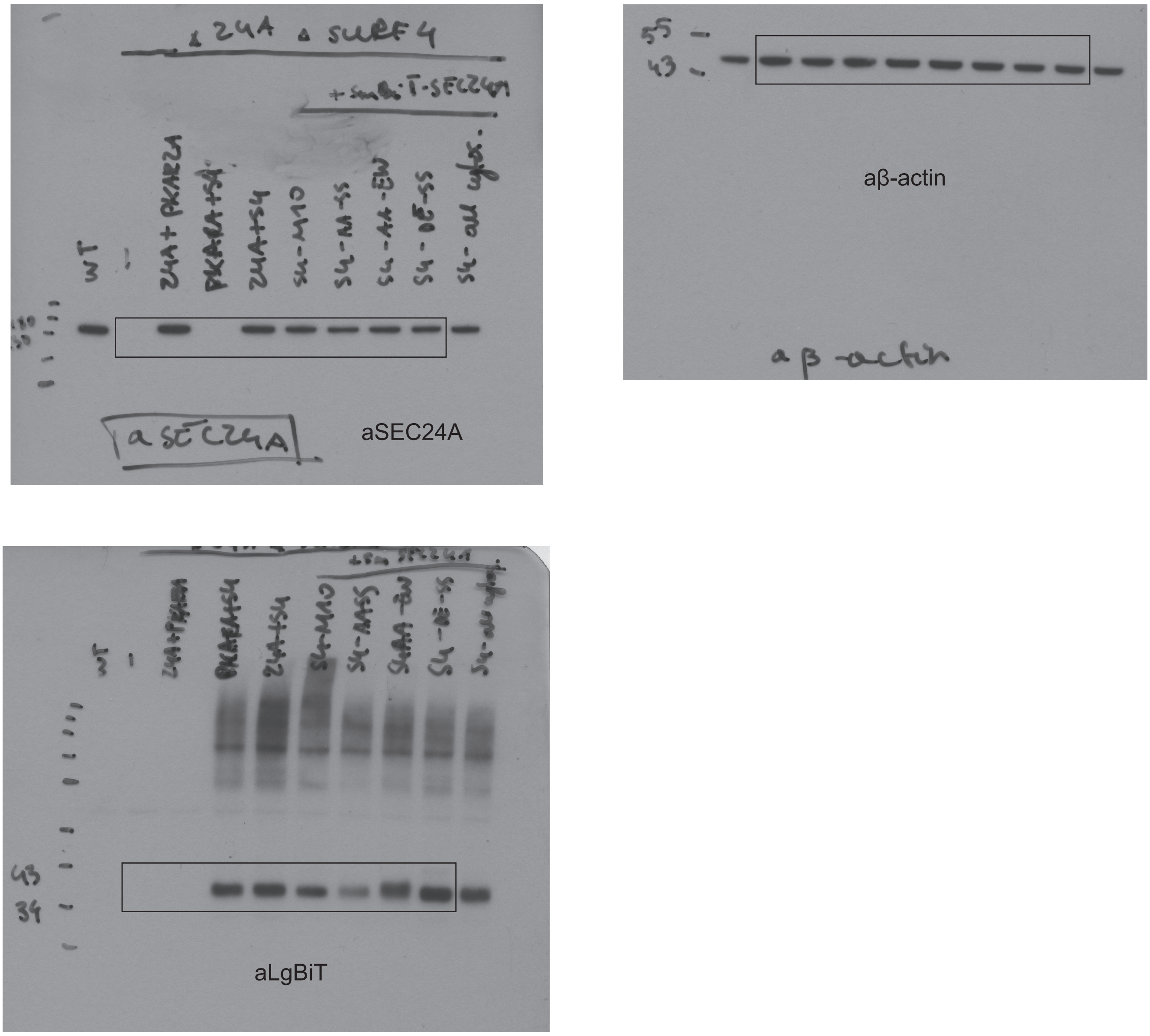

Supplement: SourceData FS1 — is the source file for Fig. S1. [file JCB_202406103_SourceDataFS1.pdf]
