# Supplementary material for: ER export via SURF4 uses diverse mechanisms of both client and coat engagement
Source: J Cell Biol. 2024 Nov 12;224(1):e202406103. doi: 10.1083/jcb.202406103 (PMC11557686; doi:10.1083/jcb.202406103)
Supplement: SourceData FS4 — is the source file for Fig. S4. [file JCB_202406103_SourceDataFS4.pdf]

SourceDataFS4

A

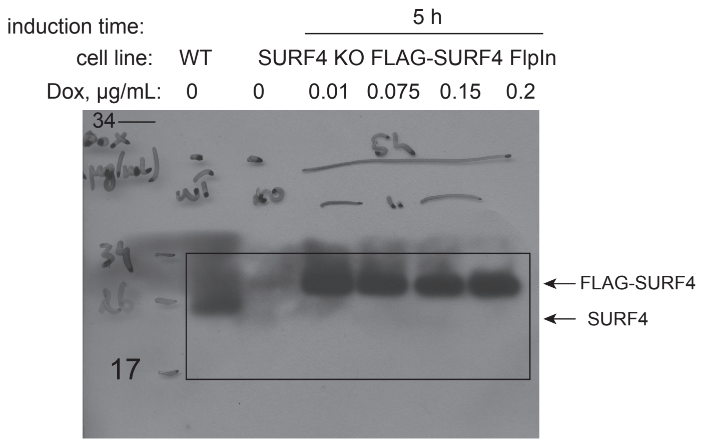

B

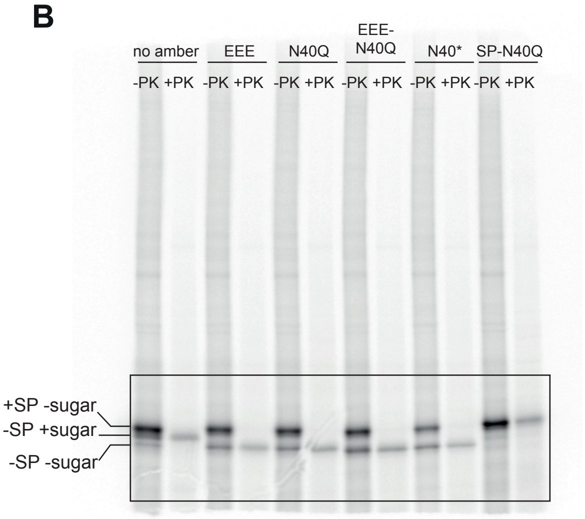

C

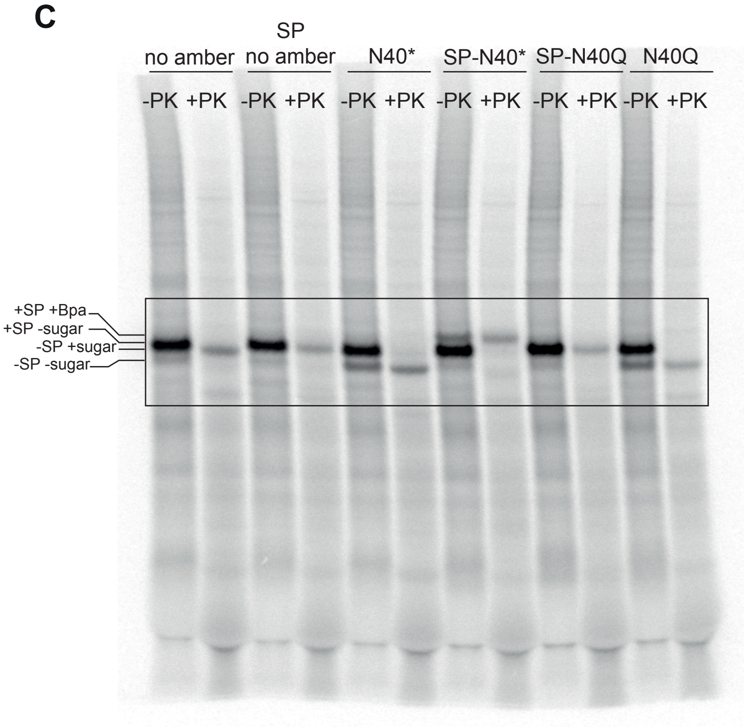

D

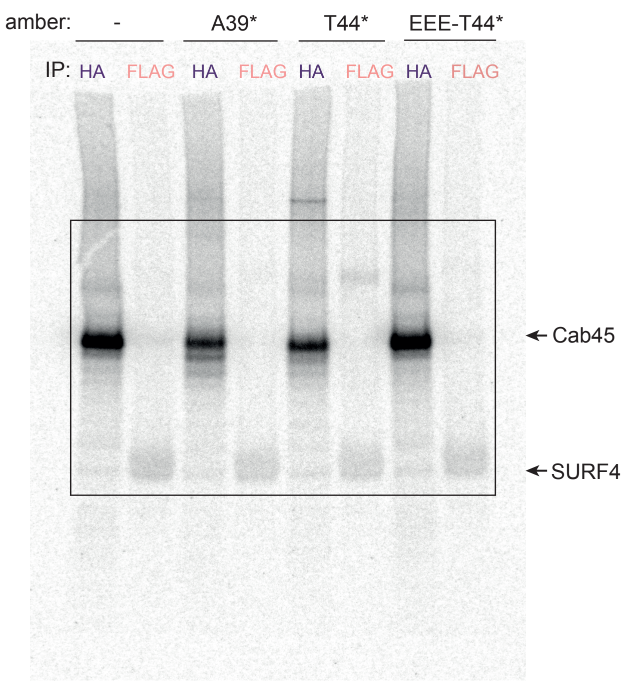

E

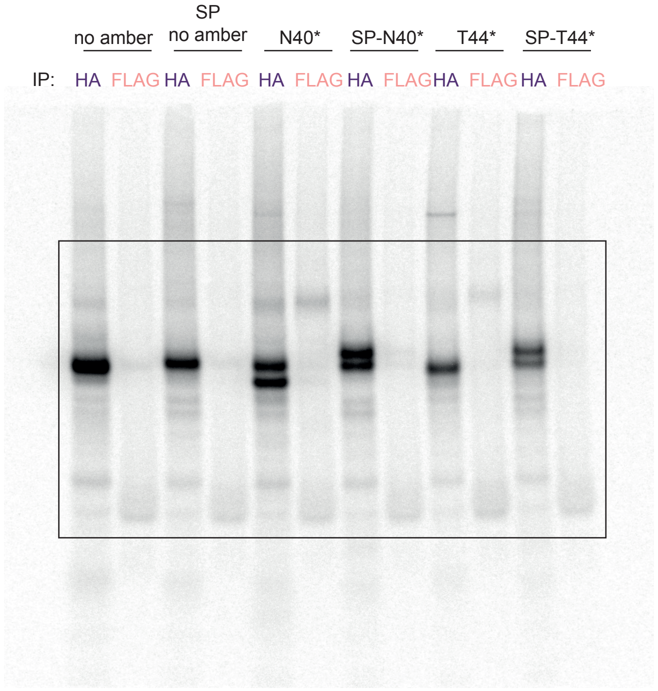

molecular weights in the main figure were obtained by aligning with the stained coomassie gel

SourceDataFS4

F

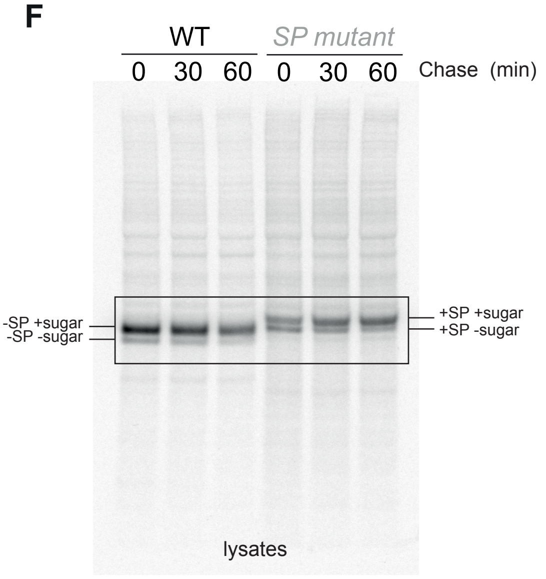

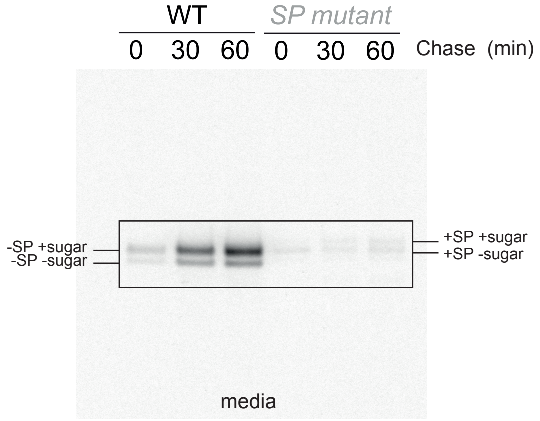

G

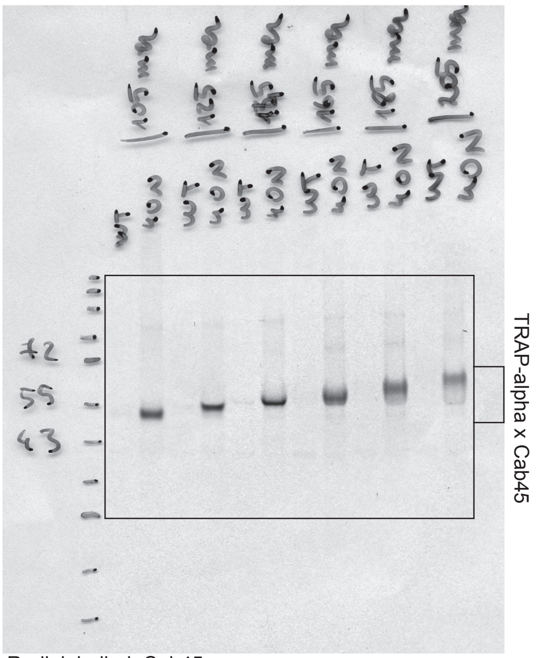

Radiolabelled: Cab45
IP: endogenous TRAP-alpha
Radioactive gel exposed to film

H

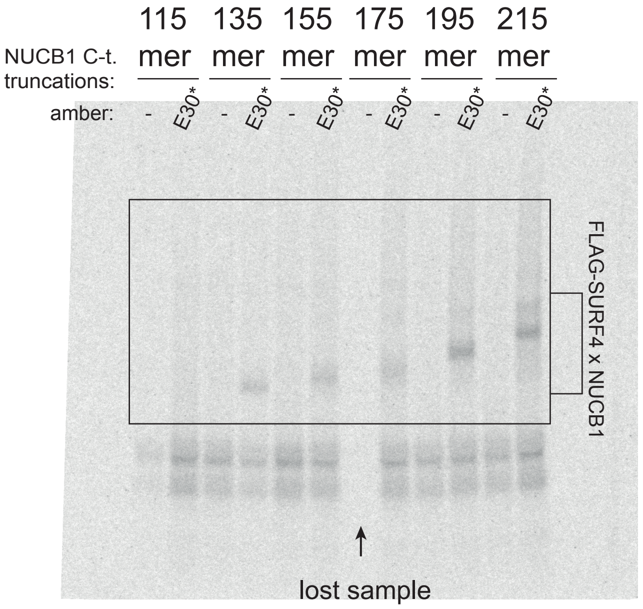

Radiolabelled: NUCB1
IP: FLAG-SURF4

molecular weights in the main figure were obtained by aligning with the stained coomassie gel
